# Involvement of the MxtR/ErdR (CrbS/CrbR) Two-Component System in Acetate Metabolism in *Pseudomonas putida* KT2440

**DOI:** 10.3390/microorganisms9081558

**Published:** 2021-07-22

**Authors:** Tania Henriquez, Heinrich Jung

**Affiliations:** Biozentrum, Mikrobiologie, Ludwig-Maximilians-Universität München, 82152 Martinsried, Germany; hjung@lmu.de

**Keywords:** two-component system, acetate utilization, *Pseudomonas putida*

## Abstract

MxtR/ErdR (also called CrbS/CrbR) is a two-component system previously identified as important for the utilization of acetate in *Vibrio cholerae* and some *Pseudomonas* species. In addition, evidence has been found in *Pseudomonas aeruginosa* for a role in regulating the synthesis and expression, respectively, of virulence factors such as siderophores and RND transporters. In this context, we investigated the physiological role of the MxtR/ErdR system in the soil bacterium *Pseudomonas putida* KT2440. To that end, *mxtR* and *erdR* were individually deleted and the ability of the resulting mutants to metabolize different carbon sources was analyzed in comparison to wild type. We also assessed the impact of the deletions on siderophore production, expression of *mexEF-oprN* (RND transporter), and the biocontrol properties of the strain. Furthermore, the MxtR/ErdR-dependent expression of putative target genes and binding of ErdR to respective promoter regions were analyzed. Our results indicated that the MxtR/ErdR system is active and essential for acetate utilization in *P. putida* KT2440. Expression of *scpC*, pp_0354, and *acsA-I* was stimulated by acetate, while direct interactions of ErdR with the promoter regions of the genes *scpC*, pp_0354, and *actP-I* were demonstrated by an electromobility shift assay. Finally, our results suggested that MxtR/ErdR is neither involved in regulating siderophore production nor the expression of *mexEF-oprN* in *P. putida* KT2440 under the conditions tested.

## 1. Introduction

Bacteria can colonize diverse environments, thanks to the development of different adaptive responses. Often, two-component systems sense changes in the environment and trigger a cellular response and adaptation. These systems consist of a sensor kinase responsible for stimulus sensing and signal transduction through autophosphorylation/phosphotransfer, and a response regulator that stimulates expression of target genes or performs other activities after phosphorylation [1]. The genome of the soil bacterium *Pseudomonas putida* KT2440, for example, is predicted to encode about seventy-two-component systems (https://mistdb.com/genomes/GCF_000007565.2 (accessed on 21 May 2021)) [2]. This Gram-negative rod-shaped bacterium belongs to the heterogenous *Pseudomonas* genus [3] that is known for its high adaptive ability [4] and a high number of two-component systems [5]. One of the roles of the two-components systems in *Pseudomonas* species is to regulate the consumption of different substrates. For example, the CbrA/CbrB system is involved in controlling the metabolism of basic amino acids (histidine, lysine, arginine) and in catabolite repression in several species such as *Pseudomonas aeruginosa* PAO1 [6,7], *Pseudomonas fluorescens* [8], and *P. putida* [9,10]. Another system involved in regulating cell metabolism is the MxtR/ErdR system, also called CrbS/CrbR, which was first described in *Vibrio cholerae* as important for acetate consumption and virulence in a *Drosophila melanogaster* model [11,12,13]. Later on, homologs of the two-component system were shown to play a role in acetate utilization also in *P. aeruginosa* [12,14], *Pseudomonas entomophila* [12], and *P. fluorescens* [15]. Sequence comparisons established a conserved nucleotide sequence in the promoter regions of MxtR/ErdR targets that was termed *acetate utilization* (ACTU) motif [15]. The results agree with earlier studies implementing the response regulator ErdR in a regulatory network controlling ethanol and acetate utilization in *P. aeruginosa* [16,17]. In addition, the sensor kinase MxtR was linked to the regulation of different virulence factors in *P. aeruginosa* PAO1 [14]. In the latter report, interruption of the gene coding for the sensor kinase, *mxtR*, affected the expression of several genes coding for the sigma factor PvdS (regulates the production of the siderophore pyoverdine, among others) and the RND transporter MexEF-OprN [14]. Additional studies on a role of homologs of the MxtR/ErdR system in regulating siderophore production and *mexEF-oprN* expression have not been reported yet.

We wondered whether the physiological function of the MxtR/ErdR system in *P. putida* KT2440 would be linked to the regulation of virulence/colonization factors, such as pyoverdine and the MexEF-OprN transporter, and/or to acetate metabolism. To answer this question, we generated a mutant for the sensor kinase (Δ*mxtR*) and for the response regulator (Δ*erdR*). Then, we analyzed the growth of these mutants in comparison to the wild type strain (WT) in minimal medium supplemented with different carbon sources. In addition, we tested pyoverdine production, MexEF-OprN activity, and the biocontrol ability (which is related to pyoverdine production and other virulence factors) of all these strains. Furthermore, we characterized the expression of genes associated with this system and analyzed the binding of the response regulator to their promoter regions. Our results indicated that the MxtR/ErdR system is not involved in the regulation of pyoverdine production and *mexEF-oprN* expression under the conditions tested, and it does not seem to be related to the biocontrol ability of *P. putida* KT2440. We found that MxtR/ErdR is linked to acetate utilization, similar to what was described for other *Pseudomonas* species. Additionally, our findings showed that *scpC* and pp_0354 were regulated by this two-component system. We also found that only one of the three genes predicted to code for acyl- or acetyl-CoA synthetases in our strain was expressed in the presence of acetate, although we were not able to locate a binding site for ErdR in its promoter region. On the other hand, we found a binding region in the promoter for the *actP-I* operon, indicating its regulation by the two-component systems. Finally, the results point out the importance of determining the actual function of these genes in the metabolism of acetate in *P. putida* KT2440.

## 2. Materials and Methods

### 2.1. Bacterial Strains and Culture Media

The strains were cultured in LB (lysogeny broth) and stored in glycerol stocks at −80 °C. A complete list of strains and plasmids can be found in Table 1 and a list of primers in Table S1. For experiments with different carbon sources (20 mM glucose, 20 mM succinate, 20 mM acetate and 0.2% *v*/*v* ethanol), minimal medium was prepared containing 1x M9-salts, 18.7 mM NH_4_Cl, 0.2 mM CaCl_2_, 2 mM MgSO_4_, and trace elements (134 µM Na_2_-EDTA, 31 µM FeCl_3_, 6.2 µM ZnCl_2_, 0.76 µM CuCl_2_, 0.42 µM CoCl_2_, 1.62 µM H_3_Bo_3_, 0.081 µM MnCl_2_). For complementation experiments, 1 mM IPTG was used. For susceptibility testing, Mueller Hinton (Sigma, St. Louis, MO, USA) medium was prepared according to the manufacturer’s instructions. For inhibition assays, PDA (potato dextrose agar, Merck) was used to promote the growth of *Fusarium oxysporum.* For growth experiments, *Pseudomonas* strains were cultured at 30 °C and *F. oxysporum* at 26 °C.

### 2.2. Generation of Mutants and Complemented Strains

Knockout strain for *mxtR* and *erdR* were generated by homologous recombination based on the pNPTS138-R6KT suicide vector according to the protocol of Lassak and colleagues [23]. Briefly, upstream and downstream regions of the gene were cloned into the vector and used to transform the wild type strain. The second recombination was induced by growing the strain in cetrimide plus 5% sucrose at room temperature for 48 h. Finally, the resulting bacteria were screened by PCR, and the respective gene deletion was confirmed by sequencing of the region containing the deletion. For complementation experiments, genes were cloned into the pSEVA224 vector [20] using the EcoRI and SmaI sites.

### 2.3. Growth Experiments

Overnight cultures in LB medium were used to inoculate 100 μL of minimal medium, with the corresponding carbon source, in a 96-well plate (Corning Costar 96-Well Black). The initial OD_600_ was adjusted to ~0.1. Plates were incubated at 30 °C in a CLARIOstar Plus (BMG LABTECH^®^) with continuous shaking, and the OD_600_ was measured every 30 min. For complementation, strains containing pSEVA224, pSEVA224-*mxtR* or pSEVA224-*erdR* were grown overnight in LB plus 50 μg/mL kanamycin and used to inoculate 2 mL of minimal medium with 20 mM acetate (OD_600_ ~0.1). Incubation was performed at 30 °C with continuous shaking (177 rpm). Later, the OD_600_ was measured after 20 h of growth. The strains were analyzed in triplicate.

### 2.4. Luciferase Assay

Transcriptional fusions were generated by cloning the promoter region of pp_4243 (*pvdL*) into the pBBR1-MCS5-*lux* vector, resulting in the P_pp_4243_*:*:*luxCDABE.* For *mexEF-oprN* operon, a previously generated reporter (P*_mexF_::luxCDABE* [22]) was used. For the luciferase assay, overnight cultures of the WT strain previously transformed with the reporter vectors were grown in LB medium (100 μL) in a 96-well plate (Corning Costar 96-Well Black) with an initial OD_600_ of ~0.1. The experiment was performed with continuous shaking at 30 °C in a CLARIOstar Plus (BMG LABTECH^®^). For the final analysis, the data of the 8 h time point of the growth curve was used for normalization (highest luciferase signal). The strains were analyzed in triplicate.

### 2.5. Gene Expression by qRT-PCR

The WT strain was grown in flasks containing 10 mL of minimal medium supplemented with 20 mM succinate, pyruvate, or acetate until the cultures reached an OD_600_ of ~0.3. Then, 3 mL of the culture were centrifuged, and the pellet was used for RNA extraction with NucleoZol (Macherey-Nagel) according to the manufacturer’s instructions. The purity, integrity, and concentration of the RNA were determined by gel electrophoresis (1% agarose gel) and NanoDrop ND-1000. Degradation of DNA was achieved by DNase I treatment according to the manufacturer’s instructions (#EN0521, Thermo Scientific, Dreieich, Germany). For reverse transcription, 1 μg of RNA was used (High-Capacity cDNA Reverse Transcription Kit from Applied Biosystems). For qPCR, cDNA was diluted 1:10 and used as a sample for the iQ SYBR Green Supermix (BioRad) supplemented with the corresponding primers (Table S1). The reaction was carried out in a BioRad CFX96 real-time system (BioRad). For normalization, the *rpoD* gene was used as the reference gene, and WT grown in succinate served as the control group.

### 2.6. Colony Morphology Assay

The original protocol from Sakhtah and colleagues [24] was performed with slight modifications. Briefly, 10 μL of overnight cultures in LB were used to inoculate cetrimide or LB agar plates. The plates were later incubated at 30 °C overnight. The colonies were photographed under UV and normal light. The final image processing was performed with ImageJ and Inkscape software.

### 2.7. Susceptibility Assay

Cultures on MH plates were utilized to prepare a bacterial suspension with OD_625_ of 0.08–0.13 in saline solution. This suspension was later used to inoculate MH plates in three different directions (to get a complete lawn). Susceptibility discs (from Thermo Scientific™ Oxoid™) were placed over the plate using tweezers. For this essay, Chloramphenicol (30 μg) and ciprofloxacin (5 μg) discs were used. Finally, plates were incubated at 30 °C for 18 h. The inhibition zone was measured with a ruler. The experiment was performed three times.

### 2.8. Growth Inhibition Assay

For growth inhibition assays with *P. putida* and *P. syringae*, the protocol of Haber and Ilan was performed with several modifications [25]. Briefly, overnight cultures of WT, *ΔmxtR*, and *ΔerdR* grown in LB were used for inoculation of LB agar plates in a line format using a cotton swab and incubated overnight at 30 °C. Then, an overnight culture of *P. syringae* was utilized to make a perpendicular line until the beginning of *P. putida* growth (with a cotton swab) and incubated overnight at 30 °C. In the case of *F. oxysporum*, a previously grown fungus on a PDA plate (1-week-old) was cut (with a borer) and put onto the agar plate with *P. putida* (previously grown in a similar way to the experiments in *P. syringae*). Further, the plate was incubated for 1 week at 26 °C. For both assays, the plates were photographed under visible light, and in the case of *P. syringae*, also under UV light. The final image processing was done with ImageJ and Inkscape software.

### 2.9. Protein Purification

The gene encoding *erdR* (pp_1635) was cloned into pET16b in frame with a nucleotide sequence encoding a N-terminal 6His tag and used to transform *Escherichia coli* C41. Briefly, an overnight culture of C41 containing pET16b-*erdR* grown at 37 °C was used to inoculate 1 L of LB medium supplemented with 100 μg/mL ampicillin (initial OD_600_ of 0.1). The culture was incubated at 30 °C with continuous shaking until reaching an OD_600_ of ~0.5, then, induction was performed with 0.5 mM IPTG for 3 h. The pellet of the culture was later collected by centrifugation, washed once with 50 mM Tris-HCl buffer (pH 8) and then, resuspended in lysis buffer (50 mM Tris-HCl pH 8, 300 mM KCl, 10% glycerol, 10 mM imidazole, 0.5 mM PMSF and 2 mM β-mercaptoethanol). For cell disruption, the pellet was processed by Constant Cell Disruptor BT40/TS2/AA (Constant Systems Ltd.) (pressure: 1.35 kBar). Cellular debris were separated from the supernatant by centrifugation. Later, the supernatant was used for an affinity chromatography by Ni-NTA. To that end, resin and sample were incubated 1 h at 4 °C and then packet onto a column (Econo column, Bio Rad). The column was later washed with imidazole (10 and 30 mM), and finally, the protein (6His-ErdR) was eluted in buffer containing 50 mM Tris-HCl pH 8, 100 mM KCl, 10% glycerol, 200 mM imidazole and 2 mM β-mercaptoethanol.

### 2.10. EMSA

Primers marked with 5′-Cy5 were designed in order to amplify 140 bp of the promoters to be analyzed. An internal region of the *sodB* gene was used as a negative control. For each EMSA reaction, 2.5 ng of DNA and different concentrations of purified 6His-ErdR (0, 0.5, 1, 2, and 4 μM) were used. The EMSA buffer contained 1 mM DTT, 2 mM MgCl_2_, 10% glycerol, 10 mM Tris-HCl pH 8, 12 ng/μL sheared salmon DNA sperm. Phosphorylation of ErdR was performed in EMSA buffer plus 2 mM acetylphosphate (when needed) for 30 min at room temperature followed by a binding reaction with the corresponding DNA for 20 min at room temperature (the final reaction volume was 10 μL). Then, the samples were loaded onto 10% polyacrylamide Tris glycine gels. Electrophoresis was performed for 1 h and 30 min in 1X Tris glycine Buffer (pH 8) at 80 V and 12 mA (maximum). Visualization of the gels was carried out in a Typhoon Scanner (GE/Amersham) using Cy5 filter.

### 2.11. Statistical Analysis

The statistical software Prism 8 was used to infer the one-way ANOVA analysis (*p* < 0.05) and *T*-test. Post hoc tests were performed in order to compare the means.

## 3. Results

### 3.1. Contribution of MxtR/ErdR to the Growth of P. putida KT2440 in Different Carbon Sources

**Figure 1:**
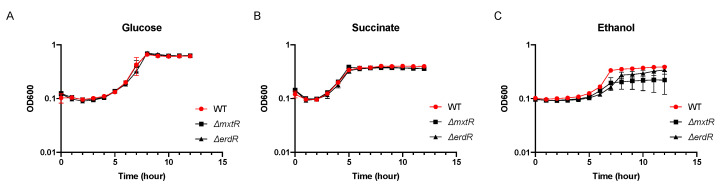

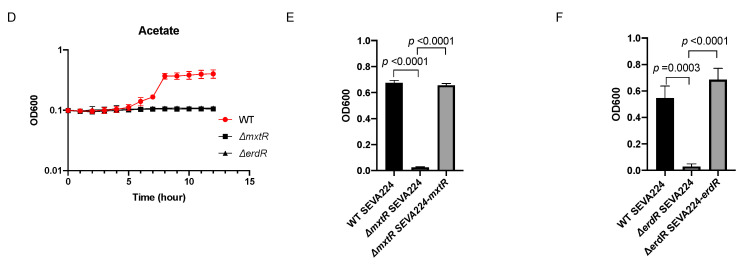
Growth phenotype of WT and the Δ*mxtR* and Δ*erdR* strains in the presence of different carbon sources. Growth experiments were performed in minimal medium supplemented with 20 mM glucose (**A**), 20 mM succinate (**B**), 0.2% *v*/*v* ethanol (**C**) or 20 mM acetate (**D**–**F**). For (**A**–**D**), overnight cultures of given strains were used to inoculate 100 μL of minimal medium in a 96-well plate with an initial OD_600_ of 0.1. The plates were incubated at 30 °C with continuous shaking and the OD_600_ was measured in a ClarioStar reader every 30 min. For (**E**,**F**), overnight cultures of the strains transformed with given plasmids were used to inoculate minimal medium with acetate plus 1 mM IPTG with an initial OD_600_ of 0.1. The cultures were incubated at 30 °C and after 20 h, the OD_600_ was measured. All the experiments were performed a minimum of three times.

The contribution of the two-component system to the metabolism of various carbon sources, and especially acetate, was analyzed. Mutants for the sensor kinase (Δ*mxtR*) and the response regulator (Δ*erdR*) were generated, and their growth in minimal medium with glucose, succinate, ethanol, or acetate as carbon source was compared with the wild type (WT) strain (Figure 1). Our results indicated that the deletion of any of the components did not significantly affect the growth of the strain when glucose or succinate was used (Figure 1A,B). Growth on ethanol of the mutants was apparently reduced compared to WT but the results were not statistically significant due to the relatively large standard deviations. (Figure 1C). However, a severe growth defect is observed in the presence of acetate, indicating that both MxtR and ErdR are essential for its metabolism (Figure 1D). Furthermore, complementation of Δ*mxtR* and Δ*erdR* corroborated these findings (Figure 1E,F). Altogether, these results indicate that the MxtR/ErdR two-component system is active in *P. putida* and its presence is essential for the utilization of acetate as a carbon source.

### 3.2. Contribution of MxtR/ErdR to the Regulation of Factors Related to Colonization in P. putida KT2440

**Figure 2:**
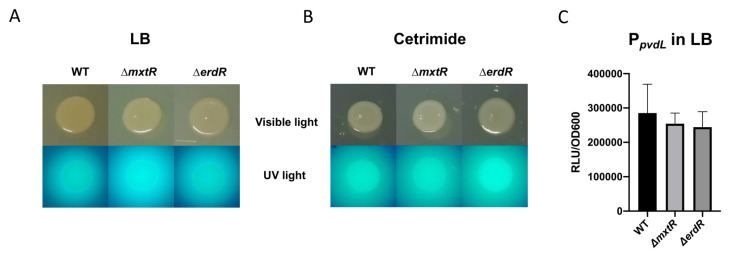

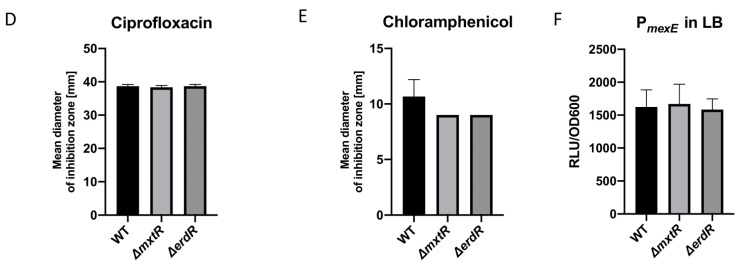
Effect of Δ*mxtR* and Δ*erdR* deletion in pyoverdine production and MexEF-OprN transporter in *P. putida.* Colony morphology assay was performed spotting 10 ul of the overnight culture into LB (**A**) and cetrimide (**B**) plates. After overnight incubation, plates were visualized and photographed under visible and UV light. The luciferase assay was performed by using WT, Δ*mxtR*, Δ*erdR* strains transformed with a plasmid containing the promoter region of the *pvdL* (**C**) and *mexE* (**F**) genes cloned upstream of the *luxCDABE* operon. The strains were grown in LB (similar to the conditions used in the work of Zaoui and colleagues [14]) in a ClarioStar reader and the Light Intensity and OD_600_ was measured every 30 min and used for normalization (data from hour number 8 of the growth curve). Susceptibility assays against ciprofloxacin (**D**) and chloramphenicol (**E**) were made through a disk diffusion method. After 18 h incubation, the inhibition zone was measured with a ruler. All the experiments were carried out at least three times.

The role of MxtR/ErdR in the regulation of pyoverdine production and expression of the RND transporter *mexEF-oprN* was investigated. The promoter regions of *mexE* (from the *mexEF-oprN* operon) and *pvdL* (coding for a non-ribosomal peptide synthase involved in pyoverdine production) were fused to the *luxCDABE* operon in a pBBR1 plasmid. In parallel, a colony morphology assay was performed to analyze pyoverdine production qualitatively. In addition, a susceptibility assay for ciprofloxacin and chloramphenicol (substrates of MexEF-OprN) was performed. Our results indicated that there was no significant difference in the size and intensity of the pyoverdine halo between WT and mutants (Figure 2A,B), and no difference in the susceptibility pattern to ciprofloxacin and chloramphenicol between the strains (Figure 2D,E). Accordingly, none of the mutants altered the activity of the promoters of *mexE* and *pvdL*, suggesting that in *P. putida* KT2440, these processes are not regulated by MxtR/ErdR (Figure 2C,F).

**Figure 3:**
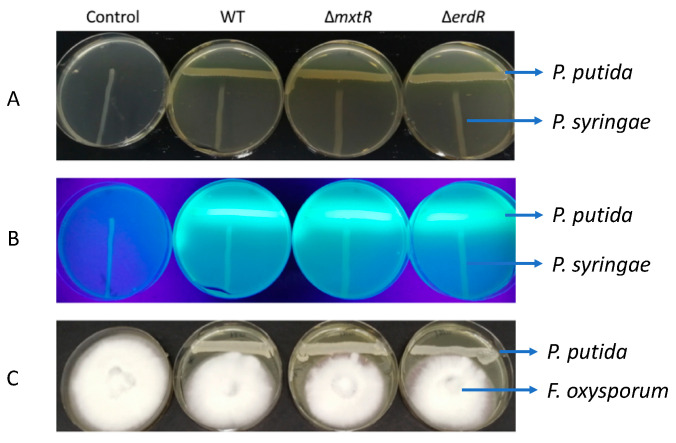
Effect of Δ*mxtR* and Δ*erdR* deletion in the biocontrol properties of *P. putida* KT2440. The inhibitory effect of WT, Δ*mxtR*, and Δ*erdR* on the growth of *P. syringae* on LB agar plates (**A**,**B**) and *F. oxysporum* in PDA agar plates (**C**) was investigated. For (**A**–**C**), from overnight liquid cultures, the *P. putida* strains were transferred in a line to a plate using a cotton swab and then incubated at 30 °C overnight. Then, for (**A**,**B**), *P. syringae* was inoculated perpendicularly to the other strain and incubated at 30 °C overnight. For (**C**), *F. oxysporum* was inoculated in the center of the plate and incubated at 26 °C for 1 week. Then, plates were photographed under visible (**A**,**C**) and UV (**B**) light. All the experiments were carried out three times.

To further investigate in a more general way whether pyoverdine or another element involved in virulence was affected, the biocontrol properties of the strain against *Pseudomonas syringae* and *Fusarium oxysporum* (two known plant pathogens) were tested. To that end, inhibition assays in LB (*P. syringae*) and PDA (*F. oxysporum*) were performed. Our results showed no significant difference in the growth behavior of the pathogen in the presence of the WT or the mutants (Figure 3), corroborating our previous findings. Altogether, these results suggest that MxtR/ErdR is not involved in the regulation of the biosynthesis of pyoverdine, MexEF-OprN, and other elements related to biocontrol activity in *P. putida* KT2440.

### 3.3. Characterization of Genes Involved in Acetate Utilization and Regulated by MxtR/ErdR in P. putida KT2440

**Figure 4:**
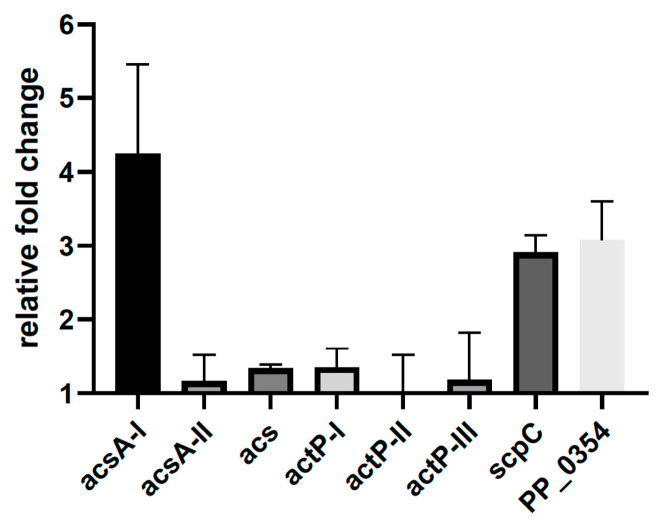
Analysis of the relative expression of genes related to acetate metabolism. Gene expression was analyzed by qRT-PCR in *P. putida* KT2440 (WT) grown in minimal medium with acetate in comparison to growth succinate as sole carbon source. The measurements were performed a minimum of three times.

Previous publications describe *acs* (acetyl CoA synthase) and *actP* (acetate transporter) as genes regulated by the homologs of MxtR/ErdR (Crbs/CrbR) in *Vibrio cholerae* [11,12,13], *P. aeruginosa* [12], *P. entomophila* [12], and *P. fluorescens* [15]. In the latter organism, a conserved DNA binding motif (ACTU motif) for ErdR was described, and besides *acs* (PFLU4766) and *actP* (PFLU1814), *scpC* (PFLU0110, putative hydrolase) and PFLU5625 (uncharacterized protein) were also identified and experimentally verified (transcriptional reporter fusions) as targets of MxtR/ErdR [15]. Since MxtR and ErdR were found to be essential for the growth of *P. putida* KT2440 on acetate, we first analyzed the expression of homologs of these genes of the strain during growth on acetate in comparison to succinate as sole carbon source. The analysis comprised the following genes: *acs* (pp_3458, putative acyl-CoA synthetase), *acsA-I* (PP_4487, putative acetyl-CoA synthetase I), *acsA-II* (PP_4702, putative acetyl-CoA synthetase II), three genes predicted to encode acetate transporters [*actP-I* (PP_1743), *actP-II* (PP_2797), *actP-III* (PP_3272)], *scpC* (PP_0154, putative propionyl-CoA:succinate CoA transferase), and PP_0354 (putative CBS domain protein). Gene expression was quantified by qRT-PCR. Our results indicated that *acsA-I*, *scpC*, and PP_0354 were upregulated during growth on acetate in comparison to succinate (Figure 4). Further bioinformatic analysis using tBLASTn and the amino acid sequence of the product of the *acs* gene of *P. fluorescens* (PFLU4766) against the genome of *P. putida* KT2440 indicated that the product of *acsA-I* has a 90% sequence identity with PFLU4766. The products of genes *acs* and *acsA-II* of *P. putida* KT2440 have 25% and 60% sequence identity with PFLU4766 (significantly lower) and were not upregulated in the presence of acetate. This observation suggests that the gene products may fulfill a different function (e.g., have a different substrate specificity) and/or are induced under different conditions. Similarly, sequence comparison of *actP* of *P. fluorescens* indicated that *actP-I* of *P. putida* KT2440 would be the closest homolog (92% identity), while *actp-II* and *actP-III* are less similar (67% and 60% identity, respectively). However, in contrast to *acsA-I*, our results showed neither an upregulation of *actP*-I nor of the other two *actP* genes of *P. putida* KT2440. Additionally, in order to test whether the lack of upregulation of *actP-I* was strictly related to the comparison with succinate, we decided to analyze its expression during growth on pyruvate (Figure S1). Our result showed that *actP-I* was downregulated with acetate in comparison to pyruvate as the sole source of carbon, suggesting that the *actP* genes are either not important for the utilization of acetate under the experimental conditions or have a different function.

**Figure 5:**
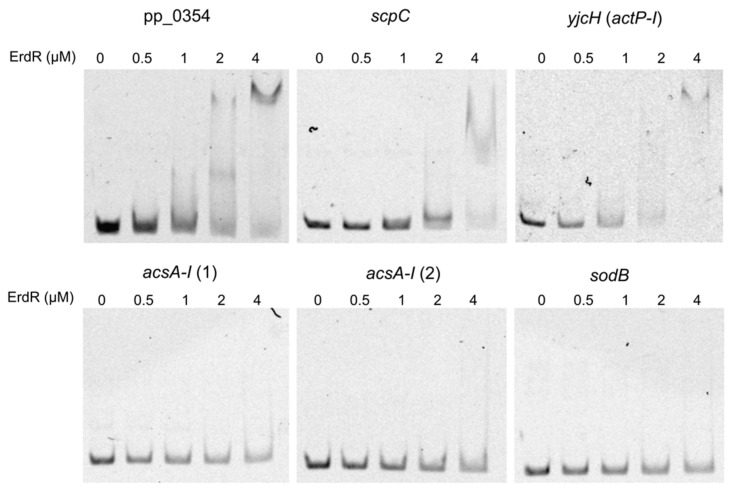
Comparison of binding of purified ErdR to promoter regions of given genes. The EMSAs were performed using different concentrations of ErdR (0, 0.5, 1, 2, and 4 μM) and 2.5 ng of the amplified promoter regions (140 bp upstream of the start codon) of pp_0354, *scpC*, *yjcH* (*actP-I*) and *acsA-I* (1). An additional part of the promoter region of *acsA-I* (from nucleotide position 122 to 261 upstream of the ATG) was also tested (*acsA-I* (2)). As negative control, an internal sequence of the *sodB* gene was used.

This approach only allowed us to link the genes to acetate metabolism in the WT strain. In order to determine the connection with the two-component system, we purified ErdR and performed an electrophoretic mobility shift assay (EMSA). In this context, we analyzed the binding of the response regulator to the first 140 bp of the promoter region of PP_0354, *scpC*, and *acsA-I.* In addition, we included in this analysis the promoter region of *yjcH* (first gene of the operon containing *actP-I)*, since, as mentioned before and according to previous studies, *actP-I* should also be under the control of ErdR. Our results showed a clear retardation in the DNA migration for PP_0354 and *scpC* promoters (Figure 5). In parallel, the analysis of the effect of acetyl-phosphate indicated that the phosphorylation of ErdR increased its affinity to DNA (Figure S2). Surprisingly, even when in our qRT-PCR experiment expression of *actP-I* in acetate medium was not stimulated in comparison to succinate medium, our results indicated the presence of a binding site in the promoter region of this operon (Figure 5). On the contrary, we did not observe a band shift dependent on ErdR with DNA fragments of the *acsA-I* promoter region (even including fragments more upstream of the start codon) (Figure 5). The result is in principle agreement with the fact that none of the fragments contained the previously predicted ErdR binding motif [15].

## 4. Discussion

Our experiments show that the MxtR/ErdR two-component system is active in the KT2440 strain and essential for the utilization of acetate as sole source of carbon. These results are in accordance with previous publications in other *Pseudomonas* species, demonstrating that the two-component system specifically controls the utilization of the short chain fatty acid [12,15].

In the initial step of acetate utilization, the short chain fatty acid is activated and acetyl-CoA is formed. Expression of the gene (*acs*) encoding the responsible acetyl-CoA synthetase was previously shown to be controlled by MxtR/ErdR in *Vibrio cholerae* and different *Pseudomonas* strains [12,13,15]. For *P. putida* KT2440, we found that the gene *acsA-I* is upregulated by acetate, whereas the other candidate genes *acs* and *acsA-II* were not. Furthermore, since the gene products of the latter two genes show much lower similarity to the acetyl-CoA synthases described for the other *Pseudomonas* strains compared with AcsA-I, it is possible that these enzymes serve different functions. In *Pseudomonas putida* U, for example, similar enzymes allow the growth on n-alkanoic acids with more than two carbon atoms, and the respective genes are also not induced by acetate [26]. Surprisingly, in contrast to other target genes, binding of ErdR to the promoter region of *acsA-I* was not detected by EMSA. Consistent with this result, the ACTU motif proposed for ErdR binding [15] is not identifiable close to the ATG. Possibly, the affinity of ErdR for the promoter region of *acsA-I* is low and therefore not detectable in the in vitro experiment, or ErdR is not involved in the regulation of the gene.

Although none of the three predicted *actP* genes of *P. putida* KT2440 was up-regulated by acetate, the band shift of the fragment containing the promoter of the *actP-I* operon indicates binding of ErdR to this region. This observation may be explained by the existence of an additional level of regulation counteracting upregulation by ErdR. Furthermore, since we compared the expression of genes in cells growing on acetate with that on succinate, we cannot exclude the possibility that one or more of the three *actP* genes are activated under both conditions. In this context, it should be mentioned that the deletion of *actP* in *P. fluorescens* does not affect growth on acetate [15]. Indeed, acetate can pass through the membrane by passive diffusion, and given the high acetate concentrations in the growth experiments, the process may be fast enough to support growth. Instead, ActP is proposed to be important for scavenging of micromolar concentrations of acetate [27]. In this context, our qRT-PCR results support the idea that *actP-I* is upregulated in the presence of low levels of acetate (probably released as byproduct of pyruvate metabolism) and repressed when not essential (such as in presence of 20 mM acetate). ActP-I, ActP-II and ActP-III belong to the solute/sodium symporter family (TC 2.A.21). Transporters of this family use an existing sodium gradient to drive the uphill transport of various solutes, such as sugars, amino acids, vitamins, carboxylic acids or ions across the membrane [28,29]. ActP-I (YjcH) shows the highest similarity (79% amino acid sequence identity) to ActP from *E. coli*, whose function as an acetate transporter was experimentally verified [27]. This was also proposed for ActP of *P. fluorescens* [15]. ActP-II and ActP-III of *P. putida* KT2440 follow with 68% and 59% amino acid sequence identity with ActP of *E. coli*. ActP-III (synonym PhaJ) is most similar (98% identical amino acids) to Ppa of *P. putida* U that was experimentally shown to function as a phenylacetate permease [30]. Similarities of ActP-I, ActP-II, and ActP-III of *P. putida* KT2440 to other members of the solute/sodium symporters are rather low (19% to 25% amino acid sequence identity with the proline transporter PutP and the vitamin transporter PanF of *E. coli*). Based on the similarities, we postulate that the three predicted ActP proteins transport acetate in micromolar concentrations (ActP-I) and/or longer fatty acids or related derivatives such as phenylacetate (ActP-II, ActP-III).

Differing from *acsA-I* and *actP-I*, both *scpC* and PP_0354 were upregulated by acetate in *P. putida* KT2440 and, binding of ErdR to their promoter regions was demonstrated by EMSA. ScpC of *E. coli* was previously shown to catalyze the transfer of coenzyme A from propionyl-CoA to succinate, and suggested to be part of a pathway that converts succinate to propionate [31]. In *P. fluorescens*, deletion of the gene (PFLU0110) impaired growth on acetate [15]. However, its precise role in the utilization of acetate remains enigmatic and requires further investigations. PP_0354 has, to our knowledge, not been functionally characterized in any strain. PP_0354 is predicted to contain a CBS domain that has been shown for other proteins to bind ligands with an adenosyl group, such as AMP, ATP, and S-AdoMet. CBS domain-containing proteins are likely to be of regulatory importance and may act as energy sensors in cells [32]. The exact function of PP_0354 needs to be explored.

While in *P. aeruginosa* PAO1 the *mxtR* gene seems to be important for the regulation of several virulence factors such as pyoverdine and the MexEF-OprN RND transporter [14], in *P. putida* KT2440 we did not observe a significant influence of the MxtR/ErdR two-component system on their regulation. Furthermore, the analysis of the biocontrol properties of *P. putida* revealed that the ability to inhibit the growth of *P. syringae* remained unaffected by the deletion of *mxtR* and *erdR*. Since inhibitory effects of *P. putida* on the growth of other microorganisms, in addition to nutrient concurrence, are mainly related to secreted factors such as pyoverdine [33] or the activity of systems such as type 6 secretion [34], our result support the lack of involvement of MxtR/ErdR in the regulation of colonization factors under the conditions tested. Consistent with this observation, deletion of *mxtR* or *erdR* did not affect virulence of *P. aeruginosa* and *P. entomophila* in a Drosophila melanogaster infection model [12].

Taken together, the results presented here assign a specific function to the two-component system MxtR/ErdR in regulating cell metabolism when acetate is used as a carbon source.

## Figures and Tables

**Table 1:** List of strains and plasmids used in this study.

**Name (Strains)**	**Description**	**Source**
Wild type (WT)	*Pseudomonas putida* KT2440	[18]
Δ*mxtR*	Derived from WT strain by deletion of pp_1695	This work
Δ*erdR*	Derived from WT strain by deletion of pp_1635	This work
*Pseudomonas syringae*	*Pseudomonas syringae* van Hall 1902. Type strain.	DSM 10604
*Fusarium oxysporum*	*Fusarium oxysporum* Schlechtendahl: Fries. Type strain	DSM 62297
*Escherichia coli* C41	BL21(DE3)-derived strain for protein overexpression	[19]
**Name (plasmid)**	**Description**	**Source**
pSEVA224	Km^R^; pSEVA221 derivative with *lacI^q^/Ptrc* expression system	[20]
pSEVA224-*mxtR*	pSEVA224 derivative with pp_1695 cloned into the multicloning site	This work
pSEVA224-*erdR*	pSEVA224 derivative with pp_1635 cloned into the multicloning site	This work
pBBR1-MCS5-*lux*	pBBR1-based plasmid containing promoter-less *luxCDABE*, and the *aacC1* gene (Gen^R^)	[21]
pBBR1-MCS5-*P_mexE_::lux*	pBBR1-MCS5-lux derivative containing *P_mexE_::luxCDABE*	[22]
pBBR1-MCS5-*P_pp_4243_::lux*	pBBR1-MCS5-lux derivative containing *P_pp_4243_::luxCDABE*	This work
pET16b-*erdR*	pET16b derivative containing pp_1635 (*erdR*)	This work

## Data Availability

The datasets generated for this study are available on request to the corresponding author.

## Supplementary Materials

The following are available online at https://www.mdpi.com/article/10.3390/microorganisms9081558/s1, Table S1: List of primers used in this study; Figure S1: Analysis of the relative expression of *actP-I*; Figure S2: Comparison of binding of purified ErdR to promoter region of pp_0354 in the absence and presence of acetyl-phosphate.
