# Supplementary material for: Involvement of the MxtR/ErdR (CrbS/CrbR) Two-Component System in Acetate Metabolism in Pseudomonas putida KT2440
Source: Microorganisms. 2021 Jul 22;9(8):1558. doi: 10.3390/microorganisms9081558 (PMC8402216; doi:10.3390/microorganisms9081558)
Supplement: Supplementary file 1 [file microorganisms-09-01558-s001.zip › microorganisms-1294004-supplementary.pdf]

Supplementary Material

Involvement of the MxtR/ErdR (CrbS/CrbR) two-component system in acetate metabolism in *Pseudomonas putida* KT2440

Tania Henriquez[1, *] and Heinrich Jung[1]

[1] Biozentrum, Ludwig-Maximilians-Universität München, Martinsried, Germany
* Correspondence: T.Henriquez@bio.lmu.de

Table S1. List of primers used in this study.

Name	Sequence (5′-3′)	Description
del1635 A_S	CCACCAGCGTTTGCATA	Deletion pp_1635
del1635 A_A1	TTGTTGCCAATGCTTGCGACTT	Deletion pp_1635
del1635 B_S1	AAGCATTGGCAACAACTTGAATC	Deletion pp_1635
del1635 B_A	AAACGGCGATCATGATT	Deletion pp_1635
check 1635 F	GATCATTTCTGTACGGCA	Checking deletion pp_1635
check 1635 F	AGCGAAATGCGATCGATGG	Checking deletion pp_1635
Clone1635 seva F	ACAGaATtCTTAGATCGATGGCCACAT	Cloning pp_1635
CloneH 1635 R	AAGCcTcgAGCCACACGCAGT	Cloning pp_1635
pp_4487 F	cgtatcgactggatcaagc	qRT-PCR
pp_4487 R	CAGTTGGAGGAAACGTTCA	qRT-PCR
actP F	CCAAACGCAACAAGTCGG	qRT-PCR
actP R	GGAAATACCCAGGAACGAG	qRT-PCR
pp_0154 F	CCTGACCATGTACTCCGAAG	qRT-PCR
pp_0154 R	CGAACACATCGGCATTGC	qRT-PCR
pp_0317 F	CCAACGCGATACCCTCGAT	qRT-PCR
pp_0317 R	GGTCGAATGCTGCCTGTTC	qRT-PCR
acs F	ATCCTGGTCAACATCAACC	qRT-PCR
acs R	AATCGGACGTCTTGAATGC	qRT-PCR
acsA-II F	TGAAACAGAACGTCGACAA	qRT-PCR
acsA-II R	CATCCTTCGTCGCTTCGTG	qRT-PCR
actpII F	GATGGCTACATCTACCTC	qRT-PCR
actpII R	ATACGAGGTGATATCGGCAA	qRT-PCR
actP-III qPCR F	CAGTGATGCCAAGGAAGC	qRT-PCR
actP-III qPCR R	TGCCGACCATGACGATG	qRT-PCR
Clone mxtR F2	TTTGaaTtcGAGTCTTCATGTCGTTGT	Cloning pp_1695
Clone mxtR A	TGCCCGAGGTAGCGGTTACT	Cloning pp_1695
Del1695_As_BamHI	GGTGGTGGTGGATCCCAAGGTCAG	Deletion pp_1695
Del1695_Aas_OL	CTTCTATGGGGACTACCTGGCCAAAC	Deletion pp_1695
Del1695_Bs_OL	GGTAGTCCCCATAGAAGGCGATGGCG	Deletion pp_1695
Del1695_Bas_NheI	AGTTCACCGGCTAGCTGTCCCAGTACC	Deletion pp_1695
Del1695_check_s	AAGGGCCGCGAATACCAGAC	Checking deletion pp_1695
Del1695_check_as	ATCGTCGGCTGGTGTGCTTTAC	Checking deletion pp_1695
sodBF EMSA	AACACCTATGTCGTGAACCT	EMSA
sodBR EMSA	TTCCAGTAGAAGGTGTGGTT	EMSA
ScpcF EMSA	TCTGCCGGGGCTTTTCTTT	EMSA
Pscpc R	GGACTCGAGTCTCGGGCTACTGA	EMSA
0354R EMSA	TCATAGACTTACGTCCAAT	EMSA
Ppp_0354 F	TAGGCGTCCTTTTTGCTCAT	EMSA
acsAIF EMSA	AATCGCCTTGCCGTTGCT	EMSA
acsAIR EMSA	TTACAGCCTTGCCGACGAAA	EMSA
acsA-I F EMSA2	TTACCTGTCTAAGGTCGTG	EMSA
acsA-I R EMSA2	AGCAACGGCAAGGCGATTA	EMSA
actPIF EMSA	CGCGAACGGGTCATCACTT	EMSA
actPIR EMSA	TGTTTTTGTCCTAGCAGCA	EMSA
cloneH 1635F	AGACATATGGCCACATACGAAATCCTG	Cloning pp_1635 into pET16b
cloneH 1635R	AAGCCTCGAGCCACACGCAGT	Cloning pp_1635 into pET16b

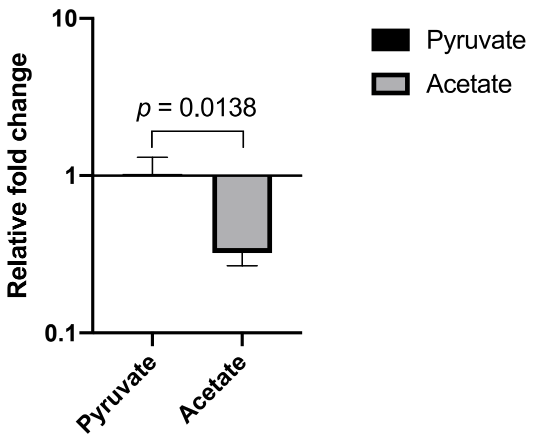

Figure S1. Analysis of the relative expression of *actP-I*. Gene expression was analyzed by qRT-PCR in *P. putida* KT2440 wild type strain grown in minimal medium with acetate compared to growth in pyruvate as sole carbon source. The experiments were performed a minimum of three times.

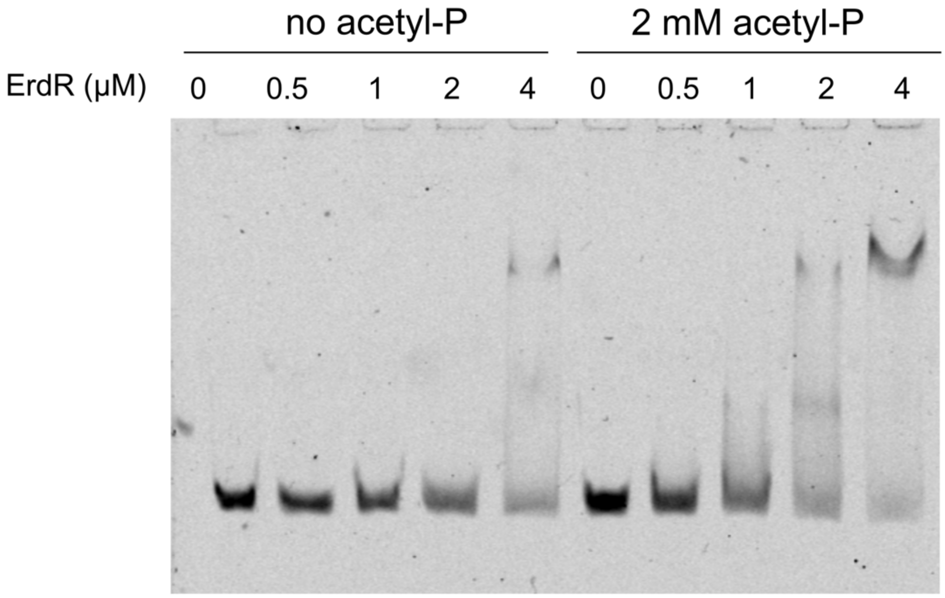

Figure S2. Comparison of binding of purified ErdR to promoter region of pp_0354 in absence and presence of acetyl-phosphate. The EMSA was performed using different concentrations of ErdR (0, 0.5, 1, 2 and 4 μM), 2.5 ng of the amplified promoter and 2 mM acetyl-phosphate (when needed).
